# Screening for ADHD in male medium secure psychiatric services

**DOI:** 10.1192/bjo.2021.89

**Published:** 2021-06-18

**Authors:** Scott Broadhurst, Kathryn Swindell

**Affiliations:** The Spinney Hospital

## Abstract

**Aims:**

Roughly 25% of the prison population are known to meet the criteria for attention-deficit/hyperactivity disorder (ADHD), a five-fold increase on the general population. Medium secure psychiatric services receive a high percentage of referrals from the prison service. ADHD has primary symptoms of inattention, hyperactivity and impulsivity. Untreated ADHD could clearly have a detrimental impact on the effectiveness of therapeutic interventions, as well as increasing incidents of violence, aggression and other transgressive behaviours.

There are two aims: To screen the medium secure services population at the Spinney Hospital, Atherton, UK for ADHD, using a validated screening tool. This would generate candidates for further structured clinical assessment for ADHD; To implement ADHD screening as a feature of the Admission Care Plan within medium secure services at the Spinney.

**Method:**

The study population is the medium secure service at The Spinney Hospital, Atherton. At the time of study this was 52 male service users.

The team members have evaluated several screening tools. The tool eventually chosen was the B-BAARS, which is a simple 6-question tool that is validated for use in adults. The tool takes around 1 minute to complete. All 52 service users were screened between 20/01/2021 and 30/01/2021.

**Result:**

1 of the 52 service users had a current diagnosis of ADHD and was being treated with medication. 3 of the 52 service users had childhood diagnoses of ADHD that had lapsed in adulthood and who were untreated. Of the remaining 51 service users without a current diagnosis of ADHD, 9 were positive on screening as worthy of further assessment (17.65%). Assessments of the 9 service users positive in screening will be completed by medical and psychology disciplines.

**Conclusion:**

There appears to be clear merit for routine screening for ADHD within medium secure psychiatric services, given the service user population and the results described above. As a result of this survey, within The Spinney Hospital the B-BAARS will be incorporated into the Admission Care Plan of all new admissions to medium secure services as a Quality Improvement Intervention. Over time this will be re-audited and there will be assessment of any impact on incidents and positive engagement with activities.

